# Celiac Disease Masking Duodenal Adenocarcinoma: A Case Report and Literature Review

**DOI:** 10.7759/cureus.68845

**Published:** 2024-09-07

**Authors:** Ahmad Salhab, Mosa R Abu Sabha, Ahmad G Hammouri, Hasan Arafat

**Affiliations:** 1 Internal Medicine, Faculty of Medicine, Al Quds University, Jerusalem, PSE; 2 Radiology, Al-Ahli Hospital, Hebron, PSE; 3 Internal Medicine, Augusta Victoria Hospital, Jerusalem, PSE

**Keywords:** celiac disease (cd), duodenal adenocarcinoma, non-responsive celiac disease, open duodenal biopsy, small bowel adenocarcinoma, upper gastrointestinal malignancy

## Abstract

Small bowel adenocarcinoma (SBA) is an uncommon gastrointestinal malignancy, with the duodenum being the most commonly affected site. This report describes a 33-year-old woman who initially presented with abdominal pain and vomiting. Initial imaging revealed abnormalities of the proximal jejunum. Endoscopic evaluation initially revealed celiac disease (CD); however, with disease progression, the patient developed bowel obstruction that led to surgical intervention with an open duodenal biopsy. The open duodenal biopsy confirmed the presence of duodenal adenocarcinoma (DA). This case demonstrates the diagnostic complexity of DA in the presence of concurrent CD due to overlapping presentations. Physicians must maintain a high suspicion of DA in the setting of progressive and difficult-to-treat CD, as early diagnosis and management of DA can significantly improve patient outcomes.

## Introduction

Small bowel malignancies are rare neoplasms of the gastrointestinal tract. They include small bowel adenocarcinoma (SBA), neuroendocrine tumors, soft tissue tumors, and lymphomas [1]. SBA is an extremely rare tumor, with the duodenum being the most common site for this neoplasm [2]. Celiac disease (CD) is an autoimmune disease triggered by gluten ingestion, and it is one of the predisposing factors for small bowel adenocarcinomas in addition to other autoimmune and congenital diseases. Here, we present a case of a 33-year-old woman, newly diagnosed with celiac disease and duodenal adenocarcinoma.

## Case presentation

**Figure 1:**
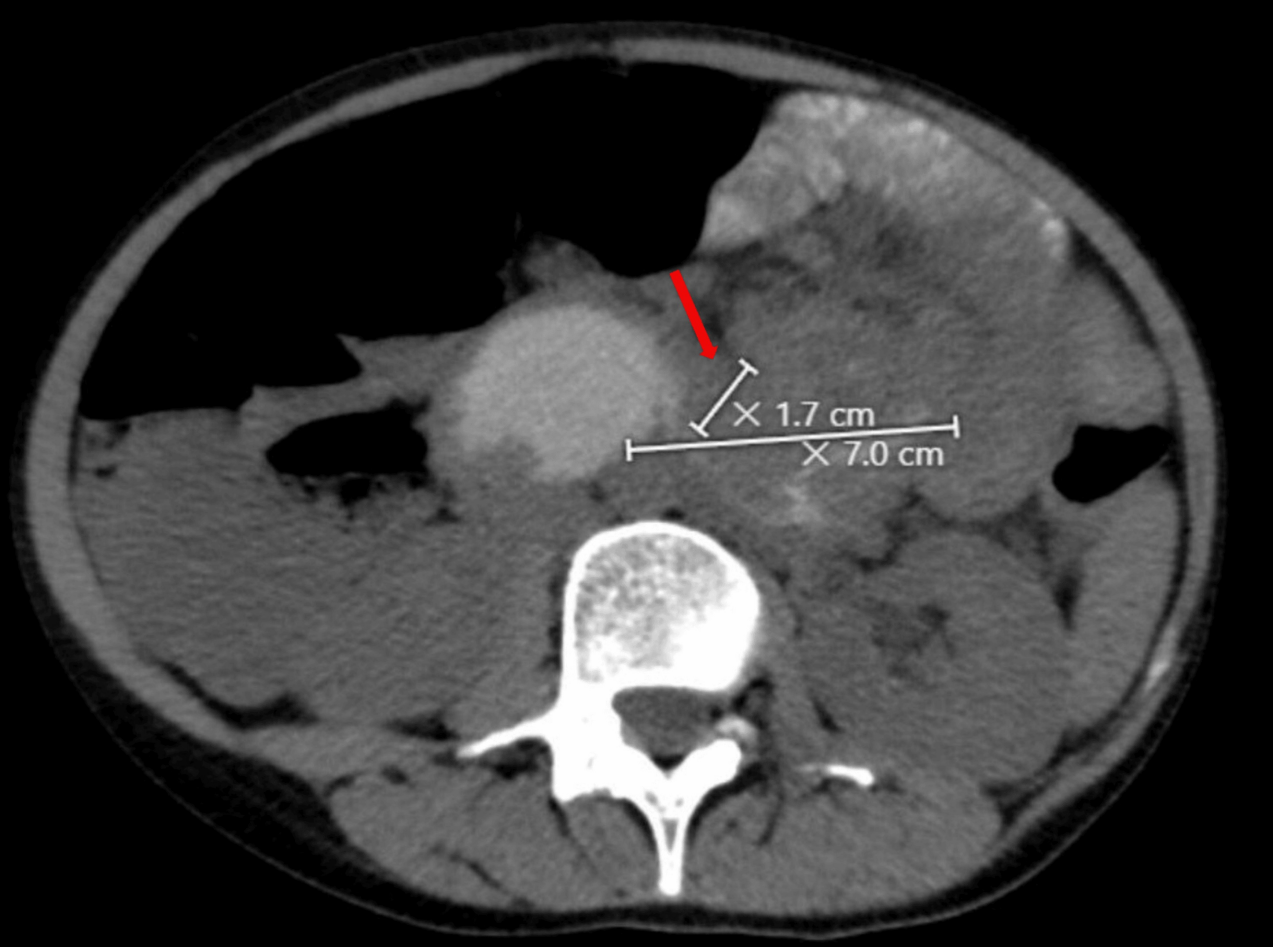
Abdominal CT scan with oral contrast showing irregular circumferential thickening involving a long segment of the proximal jejunal small bowel loops with maximum thickness measuring up to 1.7 cm.

A 33-year-old Palestinian female with no significant past medical history but a notable past surgical history, including an exploratory laparotomy with adhesiolysis, appendectomy, and bilateral lower limb explosive war injury treated with nail insertion in the left lower limb, presented with diffuse abdominal pain. This pain, radiating to the back and flanks, was colicky and associated with vomiting of gastric contents without preceding nausea, persisting for six months. Initial evaluation at a local hospital included an ultrasound, which revealed normal findings except for a 5x4 cm right ovarian cyst with minimal free fluid in the right iliac fossa. A subsequent CT scan with oral contrast demonstrated an irregular circumferential thickening involving a long segment of the proximal jejunal wall, with a maximum thickness of 1.7 cm (Figure 1). The differential diagnosis included infectious or inflammatory enteritis, atypical presentation of inflammatory bowel disease (IBD), small bowel lymphoma, and small bowel adenocarcinoma (SBA).

**Figure 2:**
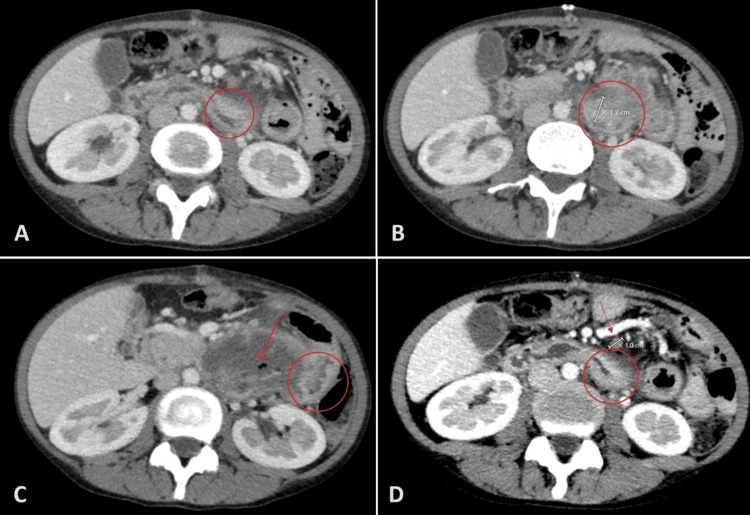
Abdominal CT scan with IV contrast showing irregular circumferential wall thickening of the proximal jejunal small bowel loops (2A, 1B), with a short segment appearing slightly dilated and fluid-filled (2C). Associated prominent regional lymph nodes are also noted measuring up to 1 cm (2D).

Despite multiple visits to the emergency department and slight pain improvement with tramadol, the patient's symptoms persisted. She reported constipation, significant weight loss, and loss of appetite but denied fever, nausea, jaundice, hematemesis, melena, or hematochezia. Family history includes a cousin with leukemia and a grandfather with unspecified gastrointestinal cancer. An upper endoscopy was unremarkable up to the duodenum. She was referred to our hospital for further evaluation and was diagnosed with partial proximal jejunal obstruction. Gastroscopy revealed a normal esophagus, a stomach with significant food content but normal mucosa, and a duodenum with decreased folds, scalloping of the second part, and narrowing in the third to fourth parts. Biopsies were obtained from the stricture and second part of the duodenum. A repeat chest, abdomen, and pelvis CT scan with IV contrast showed an irregular circumferential wall thickening of the proximal jejunal small bowel loops (Figures 2A, 2B), with a short segment that appeared slightly dilated and fluid-filled (Figure 2C). In addition, prominent regional lymph nodes measured up to 1 cm (Figure 2D).

Initial duodenal biopsies indicated lymphocytes consistent with celiac disease (CD), without evidence of lymphoma or malignancy, prompting a second opinion. A subsequent gastroscopy revealed abnormal duodenal mucosa with atrophic folds, a mosaic-like pattern, and fissuring. Biopsies confirmed lymphocytes consistent with CD and villous atrophy. During admission, the patient developed intestinal obstruction, necessitating a laparotomy with open duodenal biopsy, mesenteric lymph node biopsy, extensive adhesiolysis, and gastrojejunostomy. New duodenal biopsies indicated poorly cohesive carcinoma while lymph node biopsies showed reactive lymphadenopathy without malignancy. On oncology referral, biopsy reviews revealed one fragment with poorly differentiated adenocarcinoma and another with histological changes compatible with CD, including increased intraepithelial lymphocytes, crypt hyperplasia, and villous atrophy. Immunohistochemistry was focally positive for CDX2 and CK20 and negative for CK7.

## Discussion

Small bowel cancers are rare, accounting for only 5% of all gastrointestinal malignancies [3]. Small bowel adenocarcinoma (SBA) and neuroendocrine tumors are the two most common small bowel malignancies, both of which account for 40% of tumors [4]. The duodenum is the most commonly involved site (57%), followed by the jejunum (29%) and ileum (10%) [5].

The clinical presentation of SBA is vague, making an early diagnosis challenging. It commonly takes from 2-15 months between the onset of symptoms and the diagnosis of duodenal adenocarcinoma (DA) [6]. Around 60% of patients display symptoms at presentation, usually related to stenosis or bleeding [4]. The duodenum is the most commonly involved site in SBA (57%), followed by the jejunum (29%) and ileum (10%) [5]. Our patient initially presented with symptoms related to luminal stenosis, including abdominal pain and vomiting.

Initial evaluation of DA commonly involves upper endoscopy as well as cross-sectional imaging. Our patient initially underwent a CT scan with IV contrast, which revealed proximal jejunal enhancement with non-obstructive circumferential wall thickening just distal to the duodenojejunal junction with mesenteric lymphadenopathy. These findings were non-specific, and additional evaluation with upper endoscopy was warranted.

Upper endoscopy is a crucial diagnostic tool used to evaluate DA. In our case, although the initial endoscopy was unremarkable, persistent symptoms and significant weight loss, along with inconclusive initial findings, prompted repeat evaluations. Subsequent endoscopies with biopsies revealed duodenal lymphocytic infiltration with villous atrophy, findings consistent with CD. However, the endoscope could not advance beyond the second part of the duodenum, potentially missing a more distal pathology. This limitation, combined with the presence of CD, masked the underlying malignancy, delaying the correct diagnosis.

The diagnosis of DA requires careful histopathologic examination. Our patient's initial biopsies were only remarkable for CD. Unfortunately, due to the delay in diagnosis, our patient developed intestinal obstruction that was managed surgically, and open duodenal and mesenteric lymph node biopsies were obtained. New biopsies revealed a poorly differentiated adenocarcinoma with CD. Immunohistochemistry showed a focally positive CDX2, which is a sensitive marker for colorectal carcinoma and commonly expressed in DA [7,8].

CD is considered a major risk factor for multiple gastrointestinal tumors, particularly lymphomas and SBA [9]. Delays in diagnosis and not adhering to a gluten-free diet increase the risk of developing SBA by 60-80-fold [10]. The median onset time from diagnosis of CD ranges between 1.4 and 17 years [11]. However, CD and SBA can be diagnosed simultaneously as in our case.

Treatment options for DA are surgical resection and chemotherapy. When feasible, aggressive surgical resection offers the best outcome for these patients [12]. Oxaliplatin-based adjuvant chemotherapy is commonly offered to high-risk patients, especially those with lymph node involvement [13]. A single institution study of 122 patients who had DA with higher nodal involvement and were given adjuvant chemoradiotherapy after curative resection of DA demonstrated a similar overall survival to that of a group of patients with limited or no nodal metastases who did not receive adjuvant therapy [13]. The prognosis of DA is generally poor, with surgical resectability and the presence of metastasis at presentation being the strongest prognostic factors [14].

## Conclusions

This case demonstrates the diagnostic difficulty of DA in the context of co-existing CD. The presence of CD can mask underlying DA, delaying accurate diagnosis and appropriate treatment, especially in the presence of limited endoscopic access, which further masks the presence of an underlying malignancy. Taking this into consideration, we recommend a more thorough evaluation of CD, especially in cases where symptoms are persistent or rapidly progressive, as this might indicate the presence of an underlying DA. This approach can enhance the early diagnosis and management of DA, improving prognosis and patient outcomes.
